# Experimental data of a catalytic decolorization of Ponceau 4R dye using the cobalt (II)/NaHCO_3_/H_2_O_2_ system in aqueous solution

**DOI:** 10.1016/j.dib.2020.105463

**Published:** 2020-04-14

**Authors:** Iván F. Macías-Quiroga, Edwin F. Rojas-Méndez, Gloria I. Giraldo-Gómez, Nancy R. Sanabria-González

**Affiliations:** a Department of Chemical Engineering, Campus La Nubia, Universidad Nacional de Colombia sede Manizales, km 7 vía al Aeropuerto, Manizales AA 127, Colombia; b Department of Physics and Chemistry, Campus La Nubia, Universidad Nacional de Colombia sede Manizales, km 7 vía al Aeropuerto, Manizales AA 127, Colombia

**Keywords:** Ponceau 4R, Azo-dye, Central composite design – CCD, Bicarbonate activated hydrogen peroxide – BAP, Decolorization

## Abstract

The treatment by Advanced Oxidation Processes (AOPs) of wastewater polluted with dyes is of particular interest in the field of environmental engineering, especially for the removal azo-dyes, representing over 50% of the global annual production of dyes. Unfortunately, most azo-dyes are non-biodegradable and can be toxic to aquatic organisms. This is the first data article that applies the methodology of response surface for the optimization of decolorization of an azo-compound using cobalt in a homogeneous medium as the catalyst of a bicarbonate activated hydrogen peroxide (BAP) system which, in turn, is an emerging technology for wastewater treatment. The Response Surface Methodology (RSM) based on a Central Composite Design (CCD) was used to evaluate and optimize the influence of three experimental variables (stoichiometric dosage of H_2_O_2_, molar ratio H_2_O_2_/NaHCO_3_ and cobalt concentration) on the decolorization of Ponceau 4R. Reactions were performed at 25 °C, pH 8.3 with a reaction time of 2 h. Analysis of variance (ANOVA) showed values of R^2^ and adjusted-R^2^ of 0.9815 and 0.9648, and experimental data were fit to a second-order regression model. The optimal conditions to achieve a maximum decolorization (96.31%) of a Ponceau 4R aqueous solution of 20 mg/l were: 4.73 times stoichiometric dosage of H_2_O_2_, molar ratio H_2_O_2_/NaHCO_3_ of 1.70 and cobalt concentration of 11.16 µM. Under the optimal reaction conditions, the influence of temperature (20, 25, 30 and 35 °C) on decolorization was evaluated and data were adjusted to second order kinetics. To verify the efficiency of the BAP system on the decolorization of Ponceau 4R, under the optimal conditions of reaction, UV–Vis spectra, at different reaction times, were measured. Additionally, blank experiments in order to evaluate the effect of individual factors in the Ponceau 4R decolorization, using BAP system, were carried out. Data showed that the Co(II)-NaHCO_3_-H_2_O_2_ system is a suitable technology for the decolorization of azo-dyes aqueous solutions.

Specifications table

**Table:** 

Subject	Chemical Engineering Environmental Science
Specific subject area	Catalysis AOPs are treatment technologies designed to oxidized recalcitrant organic matter in water and wastewater through reaction with hydroxyl radicals
Type of data	Table Image Figure
How data were acquired	Oxidation reactions were performed at 25 °C for 2 h and atmospheric pressure of 78 kPa in a jacketed glass batch reactor (500 ml) under continuous stirring at 300 rpm. The reactor was loaded with 200 ml aqueous solution of Ponceau 4R at 20 mg/l and specific amounts of Cl_2_Co·6H_2_O and NaHCO_3_. The reaction started when H_2_O_2_ was added (*t* = 0). All experimental data were manually recorded. Decolorization was calculated from the initial and final concentrations of Ponceau 4R. The dye concentration was determined by using a UV–Vis spectrophotometer (Mapada UV-1200) at 507 nm wavelength. A Central Composite Design (CCD) was used to evaluate and optimize three variables on decolorization of Ponceau 4R: times the stoichiometric dosage of H_2_O_2_, molar ratio H_2_O_2_/NaHCO_3_ and cobalt concentration. 20 experiments with 3 factors and 5 levels for each factor were established. The interaction effects and optimal parameters were obtained by using a Design-Expert 8.0 software (StatEase, Inc., Minneapolis, USA). An analysis of variance (ANOVA) with 95% confidence level was carried out to identify the significance of independent variables (factors) and their interactions. The kinetic parameters of decolorization were determined by using the BAP system at four different temperatures (20, 25, 30 y 35 °C). Experimental data was fitted to a second-order model with a SciLab-6.0.2 (SciLab Entreprises SAS) function “lsqsolve”. All graphics were obtained by using an OriginPro 8.0^Ⓡ^ software (OriginLab Corporation, USA) and Microsoft Excel.
Data format	Raw Analyzed
Parameters for data collection	The effects of experimental parameters on decolorization by the BAP system were examined with CCD. 3 factors (H_2_O_2_, *n*H_2_O_2_/*n*NaHCO_3_ and evaluation of Co(II) concentration). The ranges of variables were: from 1.5 to 4.5 times the stoichiometric dosage of H_2_O_2_, molar ratio H_2_O_2_/NaHCO_3_ from 0.8 to 2.0 and cobalt concentration from 5 to 15 µM. Kinetic parameters for the decolorization under optimal condition at four different temperatures (20, 25, 30 y 35 °C) were determined.
Description of data collection	Data has information about Ponceau 4R decolorization using the Co(II)-NaHCO_3_-H_2_O_2_ system, to identify the significance and interactions of three factors of the decolorization process using a CCD. Reactions were carried out with an initial dye concentration of 20 mg/l and pH 8.3 under mild conditions (atmospheric pressure and 25 °C) for 2 h of reaction time, using the BAP system catalysed by cobalt. The supplementary material contains an Excel File with all data of Ponceau 4R decolorization using BAP system.
Data source location	Universidad Nacional de Colombia sede Manizales Manizales city Colombia 5° 01′ 45′’ N, 75° 28′ 21′’ W
Data accessibility	With the article

## Value of the data

• This is the first experimental design applying a bicarbonate activated hydrogen peroxide (BAP) system that allowed the development of an empiric model for decolorization of an azo dye (Ponceau 4R). The quadratic model obtained through RSM is adequate to predict the catalytic decolorization of Ponceau 4R in the range of experimental conditions used.
• These experimental data can be useful for the development and application of advanced oxidation processes for water and wastewater treatment, with the advantage that the BAP system is a simple, inexpensive alternative and can be used at neutral or basic pH.
• Data of the optimal decolorization conditions of Ponceau 4R can be used in equilibrium, kinetics, and mechanism studies of the oxidation of azo-dyes using the BAP system. Additionally, the use of a surface response methodology to establish the effect of variables governing the BAP system, can useful for new research on water and wastewater decolorization.
• Data will be useful to researchers and the scientific community interested in the development and application of BAP technology for the treatment of wastewater containing azo-dyes.

## 1 Data description

**Fig. 1:**
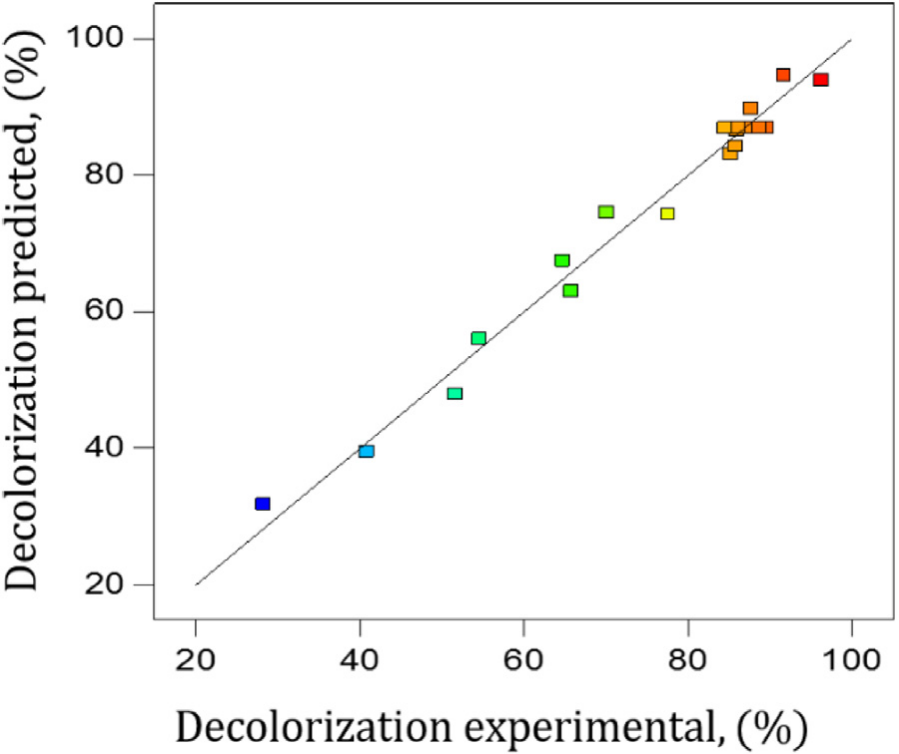
Correlation between the experimental and predicted data for decolorization of Ponceau 4R using BAP system.

**Fig. 2:**
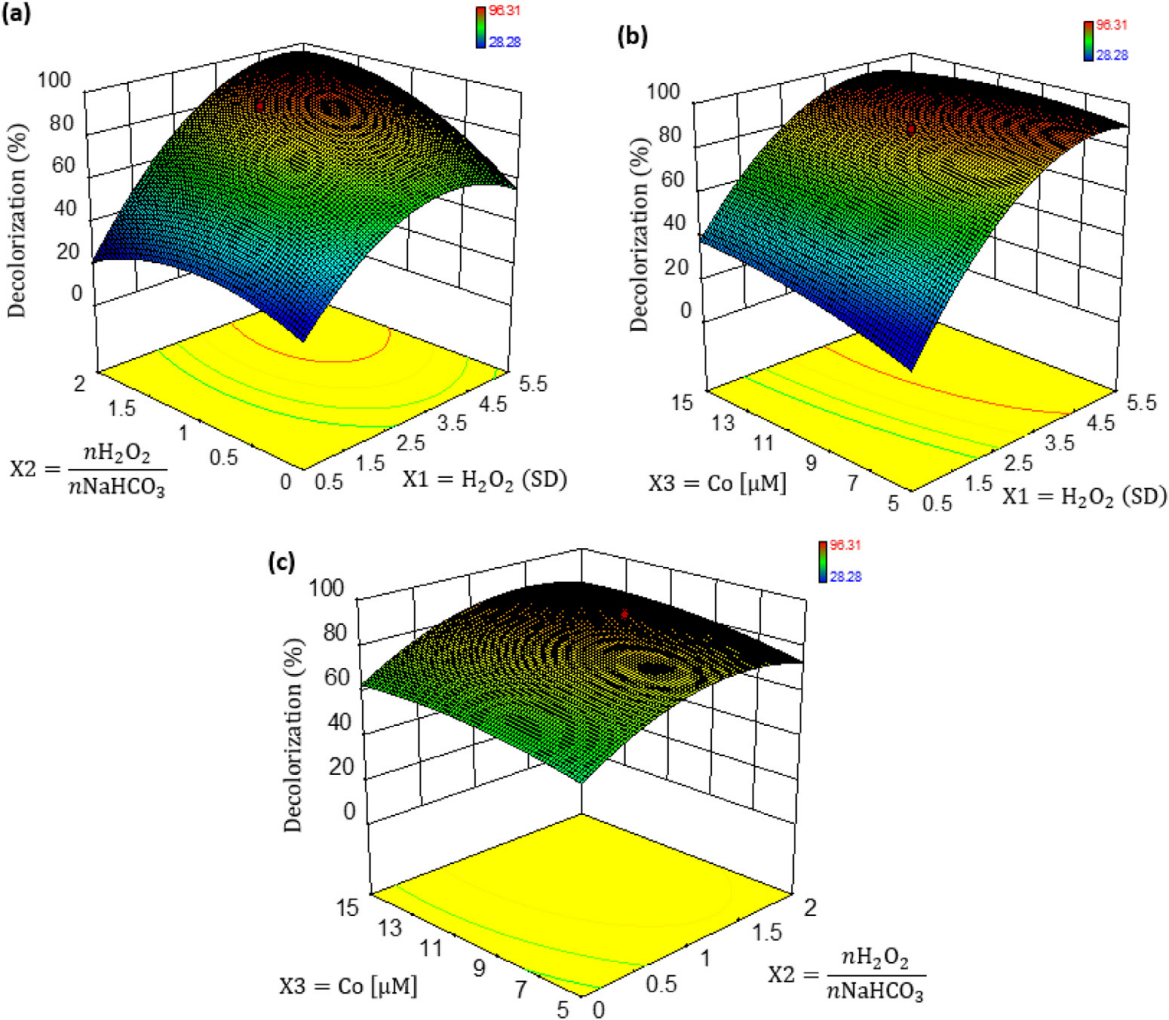
3D surface plot for interaction effect of catalytic decolorization on (**a**) H_2_O_2_ dosage vs *n*H_2_O_2_/*n*NaHCO_3_ (**b**) H_2_O_2_ dosage vs cobalt concentration (**c**) *n*H_2_O_2_/*n*NaHCO_3_ vs cobalt concentration.

**Fig. 3:**
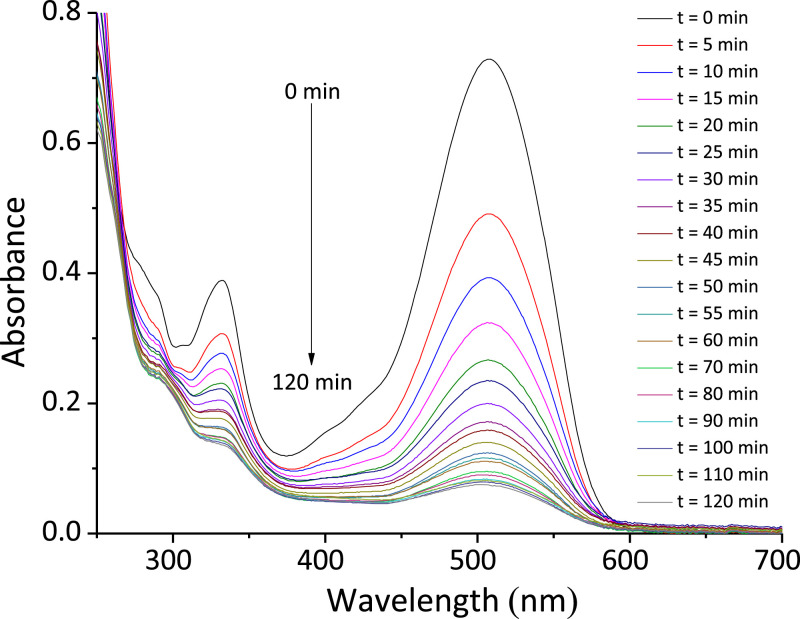
UV–Vis absorption spectra of Ponceau 4R solution during the reaction time under the optimal conditions.

**Fig. 4:**
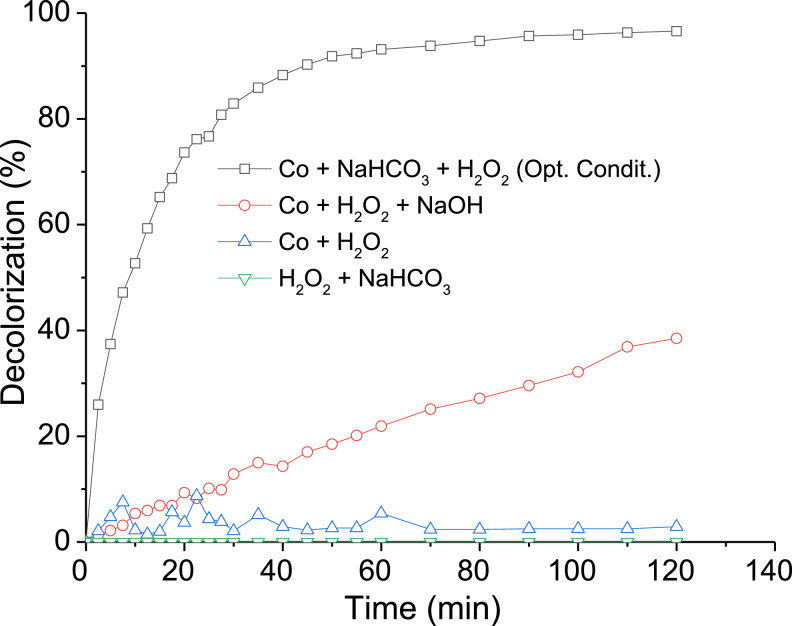
Decolorization data at optimal reaction conditions and blank tests for Ponceau 4R decolorization.

**Fig. 5:**
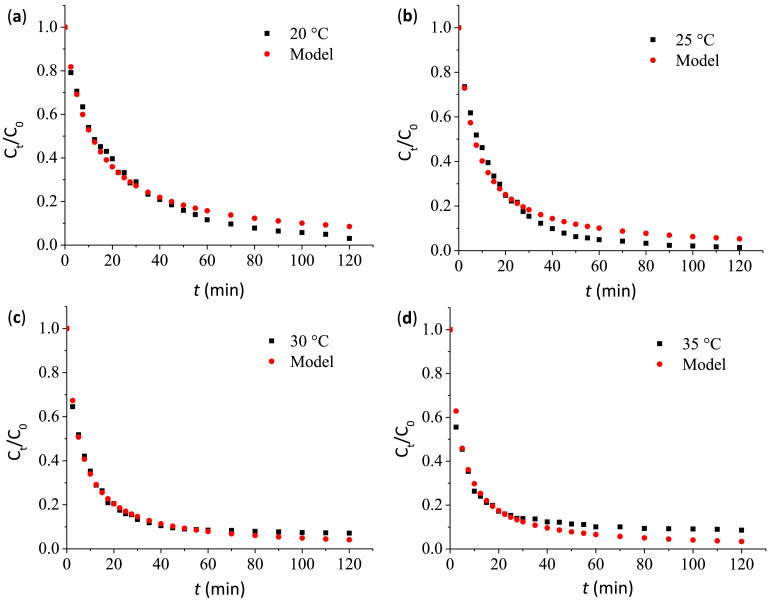
Normalized concentration of Ponceau 4R (*C_t_*/*C*_0_) as a function of reaction time (*t*) at four different temperatures (**a**) 20 °C (**b**) 25 °C (**c**) 30 °C (**b**) 35 °C.

**Fig. 6:**
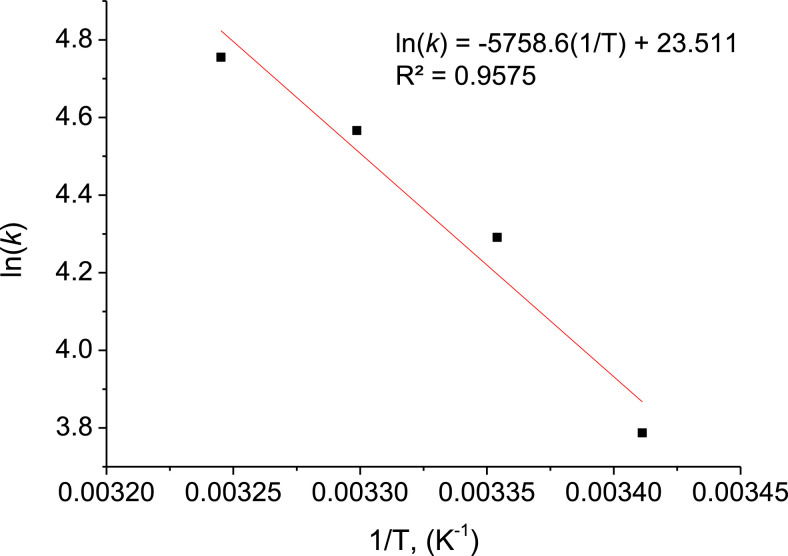
Arrhenius plot for the apparent second order rate constant.

The dataset contains eight Tables and six Figures. Data in Table 1 gives information about some properties of Ponceau 4R dye. The experimental conditions reported in literature, for the decolorization of organic colorants, using the BAP system are shown Table 2. Table 3 shows the levels of independent variables (factors) used in the experimental design for the decolorization of Ponceau 4R. The codified and experimental values of runs performed in the experimental design, with the decolorization obtained, are shown in Table 4. Table 5 summarizes ANOVA for the fitted quadratic model of Ponceau 4R decolorization. Experimental and predicted decolorization data of Ponceau 4R are shown in Fig 1. Fig. 2(a)–(c) display, by 3D graphics, the effect of interactions on the process variables of decolorization. Validation data of the empirical model for the Ponceau 4R decolorization, using the BAP system, are presented in Table 6. UV–Vis absorption spectra of aqueous solution of dye as a function of the reaction time are shown in Fig. 3. Fig. 4 illustrates the decolorization data at optimal reaction conditions and blank tests. Total organic carbon (TOC) and total nitrogen (TN) removals for decolorization of Ponceau 4R at optimal conditions and blank tests are summarized in Table 7. The monitoring of decolorization as a function of reaction time under optimal conditions, at four different temperatures are shown in Fig. 5. Fig. 6 represents Arrhenius linear relationship between ln(*k*) and 1/*T*(K). Table 8 shows the kinetic parameters of the second-order model fit and the coefficient of determination (R^2^) for the Ponceau 4R decolorization using BAP system at different temperatures.

**Table 1:** General properties of Ponceau 4R [1].

Characteristic/Property	Value
IUPAC name	Trisodium (8Z)−7-oxo-8-[(4-*sulfonatonaphthalen*-1-*yl*)*hydrazinylidene*]naphthalene-1,3-disulfonate
Synonym	Red Ponceau 4R, Acid Red 18, New Coccine, Ponceau 4 R
C.I. number	16,255
CAS number	2611–82–7
Molecular structure	
Molecular formula	C_20_H_11_N_2_Na_3_O_10_S_3_
Molar mass	604.473 g/mol
λ_max_ (nm)	507
Classification	Azo (monoazo)

**Table 2:** BAP system parameters used by other authors in the literature for the decolorization of organic colorants.

Dye	H_2_O_2_, (mM)	H_2_O_2_, (SD)	NaHCO_3_, (mM)	*n*H_2_O_2_/*n*NaHCO_3_	Co(II), (µM)	Ref.
Methylene blue	20	3	25	0.8	20	[3]
Reactive brilliant red X-3B	4	1	10	0.4	5	[4]
Methylene blue, X-3B, methyl orange, rodhamine B	10	4	10	1.0	10	[5]
Orange II	4	2	10	0.4	5	[6]
Orange II	10	0.5	2.5	4.0	5	[7]

SD = Times the stoichiometric dosage of H_2_O_2_.

**Table 3:** Levels of independent variables used in the CCD.

Independent variable	Factor coded	Level				
		−1.682	−1	0	+1	+1.682
Times the stoichiometric dosage of H_2_O_2_, (SD)	X1	0.477	1.5	3	4.5	5.523
Molar ratio of H_2_O_2_ and NaHCO_3_, (*n*H_2_O_2_/*n*NaHCO_3_)	X2	0.391	0.8	1.4	2	2.409
Cobalt concentration, (µM)	X3	1.591	5	10	15	18.409

**Table 4:** Codified and experimental values of runs performed in the experimental design.

Run Number	Codified Values			Experimental Values			Decolorization, (%)
	X1	X2	X3	H_2_O_2_, (SD)	*n*H_2_O_2_/*n*NaHCO_3_	Co(II), (µM)	
1	1.682	0	0	5.52	1.4	10	96.31
2	0	1.682	0	3	2.41	10	77.64
3	0	0	−1.682	3	1.4	1.59	64.74
4	0	0	1.682	3	1.4	18.41	85.83
5	0	0	0	3	1.4	10	84.55
6	−1	1	−1	1.5	2	5	40.85
7	0	0	0	3	1.4	10	89.66
8	0	0	0	3	1.4	10	86.13
9	1	1	−1	4.5	2	5	87.73
10	−1	1	1	1.5	2	15	54.60
11	0	0	0	3	1.4	10	87.41
12	1	1	1	4.5	2	15	91.72
13	−1	−1	−1	1.5	0.8	5	51.65
14	−1	−1	1	1.5	0.8	15	65.81
15	0	0	0	3	1.4	10	85.07
16	−1.682	0	0	0.48	1.4	10	28.28
17	0	0	0	3	1.4	10	88.73
18	1	−1	−1	4.5	0.8	5	85.28
19	1	−1	1	4.5	0.8	15	85.99
20	0	−1.682	0	3	0.39	10	70.13

SD = Times the stoichiometric dosage of H_2_O_2_.

**Table 5:** ANOVA for response surface quadratic model for decolorization of Ponceau 4R using the BAP system.

Source	Sum of square	D_f_	Mean square	F value	*p*-value
X1	4658.18	1	4658.18	378.385	<0.0001
X2	0.11	1	0.11	0.009	0.9281
X3	339.37	1	339.37	27.567	0.0004
X1X2	113.93	1	113.93	9.255	0.0124
X1X3	67.34	1	67.34	5.470	0.0414
X2X3	1.03	1	1.03	0.084	0.7783
X1^2^	1042.42	1	1042.42	84.676	<0.0001
X2^2^	279.92	1	279.92	22.738	0.0008
X3^2^	220.58	1	220.58	17.917	0.0017
Model	6517.77	9	724.20	58.827	<0.0001
Residual	123.11	10	12.31		
Lack of Fit	102.42	5	20.48	4.951	0.052
Pure Error	20.69	5	4.14

**Table 6:** Tests for the validation of the experimental design.

Experimental Values			Decolorization		Error, (%)
H_2_O_2_, (SD)	*n*H_2_O_2_/*n*NaHCO_3_	Co(II), (µM)	Predicted, (%)	Experimental, (%)	
1.98	1.56	5	58.45	61.37 ± 0.97	5.06
4.00	1.00	5	85.12	89.42 ± 0.08	5.04
4.50	1.40	5	90.86	91.94 ± 0.04	1.18
4.50	2.00	10	96.14	94.11 ± 0.30	2.11

**Table 7:** TOC and TN removals for decolorization of Ponceau 4R at optimal conditions and blank tests.

Test	Reaction conditions	Removal, (%)	
		TOC	TN
Co(II) + NaHCO_3_ + H_2_O_2_ (optimal conditions)	BAP system under optimal conditions: Co(II) concentration of 11.16 µM, 4.73 times the stoichiometric H_2_O_2_ dosage and 1.69 of molar ratio H_2_O_2_/NaHCO_3_.	13.91±1.04	19.63±0.78
Co(II) + NaOH + H_2_O_2_ (Blank test)	This test was performed in the absence of NaHCO_3_ adjusting the pH of dye solution (20 mg/l) to 8.3 through the addition of 0.1 M NaOH. Co(II) concentration and H_2_O_2_ dosage were 11.16 µM and 4.73 times the stoichiometric dosage.	0.64±0.13	1.2 ± 0.47
Co(II) + H_2_O_2_ (Blank test)	This test was performed in the absence of NaHCO_3_ (pH was not controlled). The dye solution (20 mg/l) was added with a Co(II) concentration of 11.16 µM and 4.73 times the stoichiometric dosage of H_2_O_2_.	ND	ND
H_2_O_2_ + NaHCO_3_ (Blank test)	This test was performed in the absence of Co(II). The dye solution (20 mg/l) was added with 4.73 times the stoichiometric dosage of H_2_O_2_ and an amount of NaHCO_3_ that guaranteed an *n*H_2_O_2_/*n*NaHCO_3_ of 1.70.	ND	ND

ND: Not detected.

**Table 8:** Kinetic parameters obtained after fitting for the second order model.

T(°C)	*k* (l mol^−1^ s^−1^)	R^2^
20	44.147	0.9860
25	73.051	0.9762
30	96.182	0.9951
35	116.160	0.9711

## 2 Experimental design, materials and methods

### 2.1 Materials

Ponceau 4R (89 wt%) was a reagent food-grade purchased from Retema S.A.S.-Colombia, whose properties are summarized in Table 1. A stock solution of Ponceau 4R (20 mg/l) was made up by accurately dissolving a weighed quantity of the dye in double-distilled water. Cl_2_Co•6H_2_O, NaOH and NaHCO_3_ were of analytical grade, obtained from Merck KGaA (Darmstadt, Germany), while H_2_O_2_ (30 wt%) were obtained from Sigma–Aldrich (Saint Louis, MO, USA). 100 ml a stock solution of Co(II) (4000 µM) was made up by dissolving 95.2 mg of Cl_2_Co•6H_2_O in double-distilled water, and aliquots of this solution (between 250 and 920 µl) were added to the reactor to obtain the required concentration of cobalt.

### 2.2 Catalytic decolorization tests

The decolorization catalytic reaction was performed in a batch glass reactor, open to atmosphere, thermostated at 25 °C, under constant magnetic stirring at 300 rpm. For each test, the reactor was loaded with 200 ml of aqueous solution at 20 mg/l, plus specific amounts of NaHCO_3_ and Co(II). Then, the total H_2_O_2_ dosage was added to start the reaction (*t* = 0). The dosage of H_2_O_2_ was varied in multiples of stoichiometry amount, which is theoretically required to completely oxidize one mole of Ponceau 4R into CO_2_, water (H_2_O) and mineral acids, according to Eq. (1):

(1) $${\left[C_{20}H_{11}N_{2}O_{10}S_{3}\right]}^{3-}+51H_{2}O_{2}+5OH^{-}\;\to \;59H_{2}O+20CO_{2}+2NO_{3}^{-}+3SO_{4}^{2-}$$

Decolorization was measured by monitoring the absorbance of dye in the aqueous medium at its respective maximum absorption wavelength (*λ_max_* = 507 nm), using a UV–Vis spectrophotometer (Mapada UV-1200, China). The concentration interval went from 0 to 20 mg/l, with a correlation coefficient (R^2^) of 0.9993. Detection limit (DL) and quantification limit (QL) were 0.12 mg/l and 0.36 mg/l, respectively. The decolorization was calculated from Eq. (2):

(2) $$Decolorization\;\left(\%\right)=\frac{C_{0}-C_{t}}{C_{0}}\times 100$$

where *C*_0_ is the dye concentration at *t* = 0 and *C_t_* is the dye concentration at time *t*.

### 2.3 Experimental design

The central composite design (CCD) is the most popular class of response surface design methodology used for fitting second-order models in the design of experiments [2]. The CCD was used in this work, considering the minimum and maximum levels for H_2_O_2_ (from 1.5 to 4.5 times the stoichiometric dosage -SD-), molar ratio of H_2_O_2_/NaHCO_3_ (from 0.8 to 2) and cobalt concentration (from 5 to 15 µM). The ranges considered for the three independent variables were chosen from data reported by others authors in the literature (Table 2).

Table 3 shows the description of experimental ranges and the relationship between codified and real values [8]. Low and high levels are denoted by −1 and +1, respectively, and the central points as 0. The ±α value depends on the number of variables and, for three variables, it is ±1.682 [8].

The list of the 20 experimental runs and decolorization values are shown in Table 4. The run corresponding to the central point was performed six times (run 5, 7, 8, 11, 15 and 17).

Data analysis of variance (ANOVA), using Design Expert software version 8.0 (StatEase, Inc., Minneapolis, USA) for Ponceau 4R decolorization with 95% confidence level are show in Table 5.

The quadratic model for catalytic decolorization of Ponceau 4R can be described by Eq. (3):

(3) $$\begin{array}{ccc} Decolorization\;\left(\%\right) & = & -25.7918+32.9902X_{1}+20.3570X_{2}+5.1198X_{3}+4.1930X_{1}X_{2}\\ & & -0.3868X_{1}X_{3}+0.1194X_{2}X_{3}-3.7799X_{1}^{2}-12.2423X_{2}^{2}-0.1564X_{3}^{2} \end{array}$$

The coefficients of the response model R^2^ and adjusted-R^2^ were 0.9815 and 0.9648, respectively.

Fig. 1 shows the correlation between the experimental and predicted data for decolorization of Ponceau 4R using BAP system. Fig. 2 shows the 3D surface generated by Eq. (3) and the influence of variables analyzed in the decolorization. By means mathematical optimization of the model (maximization of a Eq. (3) occurs where its derivative is equal to zero), the values of variables to achieve the maximum decolorization (98.13%) were determined, corresponding to 4.73 times the stoichiometric dosage of H_2_O_2_, 1.70 of *n*H_2_O_2_/*n*NaHCO_3_ and 11.16 µM of cobalt concentration. After carrying out the decolorization reaction under optimal conditions, a decolorization experimental of 96.46 ± 0.166% (error 1.70%) was obtained.

Additional catalytic decolorization tests, under the optimal operating conditions, were carried out in order to validate the quadratic model. The experimental and predicted values with Eq. (3) are shown in Table 6.

### 2.4 Decolorization monitoring using UV–Vis spectra

The efficiency of the Co(II)-NaHCO_3_-H_2_O_2_ system for Ponceau 4R decolorization under the optimal conditions was evaluated by measuring the changes of absorption UV–Vis spectra as a function of the reaction time, and the data are displayed in Fig. 3.

### 2.5 Blank tests

Blank tests, without H_2_O_2_, NaHCO_3_ and Co(II), were performed in order to evaluate the effect of individual factors in the Ponceau 4R decolorization (Fig. 4) under the optimal conditions of the experimental design. Blank tests descriptions are summarized in Table 7. Besides, the total organic carbon (TOC) and total nitrogen (TN) removals were estimated for each test. The TOC and TN removals were calculated using the Eqs. (4) and (5):

(4) $$TOC\left(\%\right)=\frac{TOC_{0}-TOC_{f}}{TOC_{0}}\times 100$$

(5) $$TN\left(\%\right)=\frac{TN_{0}-TN_{f}}{TN_{0}}\times 100$$

where *TOC*_0_, *TOC_f_, TN*_0_ and *TN_f_* are the TOC and TN contents at the beginning and end of the reaction (Fig. 4). The contents of TOC and TN were determined by using a TOC/TN analyzer (Multi N/C 3100, Analytik Jena AG, Germany).

### 2.6 Kinetics of decolorization

Data obtained for the normalized concentration of Ponceau 4R (*C_t_*/*C*_0_) versus time (*t*), at four different temperatures (20, 25, 30 and 35 °C), are summarized in Fig. 6. Such data were adjusted to second order kinetics [9], [10]–11], according to Eqs. (5) and (6):

(5) $$\frac{C_{t}}{C_{0}}=1-\frac{kC_{0}t}{kC_{0}t+1}=\frac{1}{kC_{0}t+1}$$

Rearranging Eq. (5):

(6) $$\frac{1}{C_{t}}-\frac{1}{C_{0}}=kt$$

were *k* is apparent second order rate constant.

Experimental data *C_t_*/*C*_0_ was fitted to the model described in Eq. (5). The values of constant *k* as a function temperature (*T*) (Table 8) were obtained by using the Levenberg-Marquardt algorithm [12]. The Scilab-6.0.2^Ⓡ^ function “lsqrsolve” that minimizes the sum of square differences between experimental and predicted values of the nonlinear kinetic function, for each temperature evaluated, was used. The dependence of *k* values from the reciprocal of absolute temperature (1/*T*) is shown in Fig. 6. The calculated apparent activation energy (E_a_) from the Arrhenius plot regression (Fig. 6) was 47.88 kJ/mol, a value similar to that obtained in the degradation of Acid Orange 7 by catalytic wet hydrogen peroxide oxidation (E_a_ = 47.30 kJ/mol) [12].

## Appendix Supplementary materials

Image, application 1

Image, application 2
